# Impact of an early 1000-day intervention for obesity prevention on adiposity and BMI at two years of age: A quasi-experimental study

**DOI:** 10.7189/jogh.13.04145

**Published:** 2023-12-13

**Authors:** Mercedes Díaz-Rodríguez, Celia Pérez-Muñoz, Jesús Carretero-Bravo, María José Santi-Cano, Pilar Carrasco-Sánchez, Cristina Barroso-Chirino, Bernardo Carlos Ferriz-Mas

**Affiliations:** 1 Department of Nursing and Physiotherapy, University of Cádiz, Cádiz, Spain; 2 Department of Biomedicine, Biotechnology and Public Health, University of Cádiz, Cádiz, Spain; 3 Clinic Management Unit (CMU) Puerto Real, Andalusian Health Service, Cádiz, Spain

## Abstract

**Background:**

The 1000-day period encompassing pregnancy and the first two years of postnatal life is critical for preventing childhood obesity. Existing interventions targeting this period have been characterised by great variability in duration, objectives, and evaluation indicators. We aimed to evaluate the impact of an intervention developed during the entire 1000-day period on body mass index and body fat percentage at two years of age.

**Methods:**

We designed a prospective, interventional, quasi-experimental study (ie, without randomisation or blinding of both groups) targeting mother-child pairs from the beginning of pregnancy up to two years of age belonging to the basic health area of Puerto Real (Cádiz). We developed and delivered an intervention from pregnancy to two years and assessed its effect.

**Results:**

The duration of breastfeeding and vitamin D supplementation increased significantly after the intervention. The intervention group showed lowed skinfolds values, a significantly lower body fat percentage, as well as a lower accumulation of factor at two years than the control group.

**Conclusions:**

The intervention has had an impact on body fat percentage at two years, potentially justified through its overall effect and the lower accumulation of early risk factors.

Childhood obesity is one of the greatest global challenges, with World Health Organization (WHO) data [1] showing that the prevalence of childhood overweight worldwide increased in the 5-19-year-old population from 4% in 1975 to more than 18% in 2016. According to the study conducted in 2017 by the Non-Communicable Disease Risk Factor Collaboration [2], Greece, Malta, Italy, Cyprus, and Spain had the highest prevalence of childhood and adolescent overweight in Europe. Despite the development of national [3] and regional strategies [4] in Spain, no significant reduction in childhood obesity has been achieved, with obesity remaining stable in 2011-2019 [5]. The ALADINO 2019 study showed that 40.5% of schoolchildren between six and nine years of age presented with overweight or obesity [5]. Most prevention trials have focused on schoolchildren or adolescents [6], when excess weight is often already established. The high percentage of overweight schoolchildren suggests the need to develop prevention interventions at an earlier age [7].

There are several critical periods related to the development of obesity in childhood [8], with the first comprising gestation and the first two years of postnatal life (1000 days). It is a period of maximum plasticity of early programming mechanisms, where the acquisition of lifestyle habits begins, during which we recognised several risk factors associated with later overweight and obesity in infancy, known as early risk factors (ERF). These include higher maternal pre-pregnancy body mass index (BMI), prenatal tobacco exposure, maternal excess gestational weight gain (GWG), and rapid infant weight gain (RWG) [9]. Other ERF, like gestational diabetes (GDM), childcare attendance, curtailed infant sleep, the introduction of solid food intake before the age of four months, duration of breastfeeding, and vitamin D deficiency, showed different associations in previous research [9-11], but are also potential risks in the development of obesity.

A systematic review [12] of RCTs during pregnancy and the first two years of postnatal life to prevent childhood obesity only found interventions based on the protein content of the formulas or behavioral ones focused on parents to be effective, although with little impact on excess weight. Another systematic review of 26 interventions during the first 1000 days [13] found those focusing on several ERF to be the most effective, including diet/maternal physical exercise during the first postnatal year, assessing z-score BMI between two and four years [14], sleep/feeding, and assessing weight-for-length at two years [15]. Two other interventions based on home visits and group sessions focused on diet, feeding practices, and physical activity, had an effect on BMI at 12 or 24 months [16,17]. Interventions are likely to require targeting multiple ERF before two years of age to have an impact on childhood obesity prevention, as studies have shown that the presence of two or more ERF in one individual was associated with an increased risk of overweight at four to six years of age [18]. Previous results from the control group in our study showed a similar cumulative effect on higher z-score BMI and body fat at ages as young as two years [19].

Thus, we observed that interventions had the greatest effect when they focused on changes in diet and physical activity behaviors in the family, when they took place at home or community group visits, when they included several ERF, and when they took place during the pre- and post-natal period [13]. Therefore, interventions should take place over the entire 1000-day period and encompass as many aspects as possible related to the risk of childhood obesity.

Most previous studies used BMI as the main indicator of an intervention’s impact either at its end or years later, with few using body fat as an exclusive indicator or combined with BMI. Two interventions, developed during gestation, evaluated the effect using z-BMI and skinfolds at six [20] and 12 months [21]. Another study [22] analysed the outcome at the end of the intervention from zero up to two years using BMI and skinfolds. To our best of our knowledge, there are no studies evaluating the effects of an intervention developed during the entire 1000-day period, targeting most early risk factors for infant obesity, and using z-score BMI and body fat percentage at two years as outcomes.

We aimed to evaluate the impact of an intervention for preventing childhood obesity developed during the entire 1000-day period and focused at the modifiable ERF during pregnancy (GWG, smoking) and first two years (RWG, duration of breastfeeding, duration of vitamin D supplementation, initiation of complementary feeding, and sleep habits). We evaluated the intervention’s impact by analysing the number of ERF accumulated in each individual and measuring the z-score BMI and body fat percentage at two years of postnatal age.

We hypothesised that a combined health care and educational intervention developed during the entire 1000-day period, targeting the gestational and postnatal modifiable early risk factors for childhood obesity, could improve BMI and body fat percentage at two years of life. Our general objective was to analyse the intervention’s impact on body composition at two years of age by comparing the body composition between the control and the intervention groups and analysing the intervention’s effect on the early risk factors of childhood obesity.

## METHODS

### Design

We designed a prospective, interventional, quasi-experimental study (ie, without randomisation or blinding of both groups) to evaluate the effect of a combined educational and health care intervention on adiposity and BMI at two years.

We could not perform randomisation because we had a small population, which might have led to contamination between the groups due to a transfer of information. In turn, we created a historical control group from the same population. We were also unable to blind the participants or the researchers conducting the intervention, but we did blind assessors in terms of effect assessment and data analysis.

### Population

We targeted mother-child pairs from the beginning of pregnancy up to two years of age within the basic health area of Puerto Real (Cádiz). This area comprised two clinical management units (CMUs): Puerto Real, with four pediatric quotas and Casines with two. There were no significant socioeconomic differences between the different groups.

The study population of the control group comprised mother-child pairs, incorporated when the child attended the health check-up at one or two years within 12 months of the start of the intervention and belonging to two of the four pediatric quotas of the Puerto Real CMU. We avoided possible contamination from the intervention by recruiting the children in the control group at the same time as the intervention was initiated in pregnant women in the intervention group. The study population of the intervention group consisted of mother-child pairs whose pregnancy began within 14 months of the start of the intervention, and whose mothers underwent complete follow-up of their pregnancy.

For both groups, we included mother-child pairs where pregnancies did not present any pathology incompatible with the study and where parents authorised the mother-child pair's participation in the study. We excluded pairs where infants presented any pathology that could significantly affect their growth.

### Sample

We estimated in the initial protocol that we would need 100 individuals in the control (two pediatric quotas) and 150 in the intervention group (three pediatric quotas).

**Figure 1:**
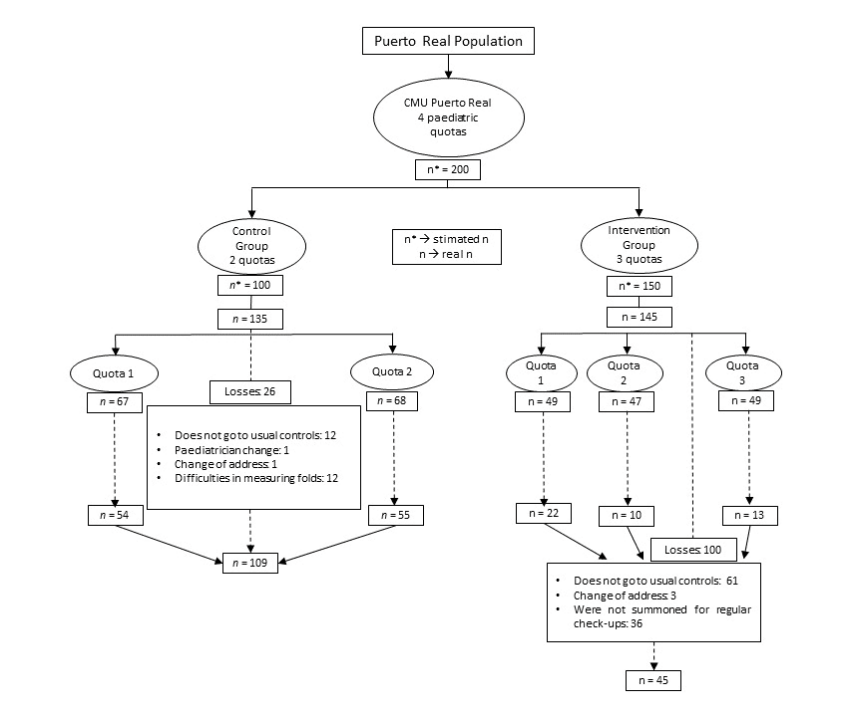
Flowchart of the intervention. Source: Prepared by the authors.

We included 135 mother-child pairs in the control group, with 26 dropping out during the follow-up period for different reasons, resulting in a final sample of 109 pairs. In the intervention group, the period of collection of weight, length and skinfolds at two years coincided partially with the COVID-19 pandemic. The temporary suspension of child health programs and the refusal of families to attend health centers limited the number of cases. This consequently led to significant drop-out and loss of participants, we obtained data from 45 pairs for the intervention group (**Figure 1**). Despite these losses, with the sample size achieved and the established significance level (0.05), we performed a post-hoc calculation of the statistical power of the mean comparison tests, resulting in an 87% power to detect a medium effect size of 0.5 [23].

### Intervention

The details of the intervention are published elsewhere [24]. In short, it centered on the use of the concept of early programming as the focus for the recommendations. In addition, we carried out the intervention in a period when parents are very receptive to their children's health, especially if the intervention itself was carried out by professionals and included in the scheduled visits, meaning it did not require any additional effort.

**Figure 2:**
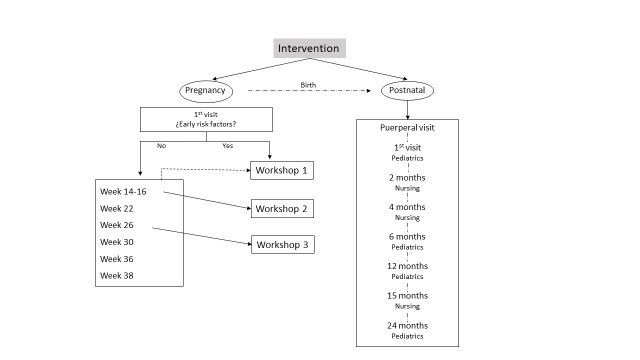
Diagram of the intervention carried out. Source: Prepared by the authors.

The intervention began at four to seven weeks of gestation up to the age of two years through the usual visits during this period (**Figure 2**). During each visit, we took every opportunity to reinforce the concept of early programming and ERF, resolve any doubts raised or observed by the professionals, and follow up on the intervention.

### Outcomes

Variables measured on the mother were: Age at delivery; BMI (kg/m2) at the beginning of pregnancy (week 4-7), classified as obesity (≥30), overweight (25-29.9), normal weight (18.5-24.9), and underweight (<18.5); GWG, classified as adequate or excessive according to the values recommended by the Institute of Medicine [25]; smoking during pregnancy, considering as smoking any amount of tobacco; GDM, by asking the mother directly; type of delivery, categorised as cesarean or not.

Variables measured in the first two years of the child were: Sex of the newborn; weight (grams) at birth and four, six, 12 and 24 months; length (cm) of the newborn; duration of exclusive breastfeeding (EBF)≥4 months; Duration of breastfeeding (BF) up to 12 months [26]; AWG according to weight z-score, adjusted for age and sex at birth and four, six, 12 and 24 months, using the Anthro program [27] and considering AWG an increase in z-score >0.67 standard deviations (SD) [28]; early introduction of complementary feeding (before four months); duration of vitamin D supplementation in the first year.

We measured the following dependent variables at two years: BMI (kg/m2) and body fat percentage using Siri's formula [29]; body density obtained from the measurement of the four skinfolds (biceps, triceps, subscapular, and suprailiac) by applying Brook's validated formulas [30].

### Data collection

We collected the variables by posing direct questions to parents during visits in the first and second year of life; meanwhile, we retrieved weight, length, and BMI data from records of the integrated healthcare management system of the Andalusian Health Service, Diraya.

We calculated the weight of pregnant women with an ADE M320000-01T scale (ADE Germany GmbH, Hamburg, Germany) with a reading range of two to 250 kg and an accuracy of 100 g and length with an ADE stadiometer, coupled to the scale, with a reading range of 60-210 cm. We determined weight at birth and four, six, 12, and 24 months with the infant undressed using a Soehnle baby scale, with a reading range of 0 to 20 kg and an accuracy of 10 g. We measured the length with the infant supine with an Añó-Sayol rigid stadiometer with a reading range of 25 to 90 cm and an accuracy of 0.5 cm. We calculated BMI at two years of age using the WHO growth standards Tables [31] and skinfolds with a Holtain Skinfold Caliper plicometer (Holtain Ltd, Dyfed, UK) with an amplitude of 0 to 46 mm, and a constant pressure of 10g/mm2. We measured the skinfolds on the left side of the body in triplicate and by a single observer for each pediatric group, according to the techniques recommended by the WHO [32]. The personnel in charge of measuring the folds received training from the same expert.

### Statistical analysis

We performed a descriptive analysis of the variables using the most common summary statistics (means, deviations, and confidence intervals for ordinal/continuous variables, frequencies and percentages for categorical variables). We checked the normality of distribution for anthropometric measurements (BMI and percentage of body fat) with the Kolmogorov-Smirnov test if the sample size of the groups was greater than 50 and with the Shapiro-Wilk test otherwise. We compared these measurements between boys and girls using the appropriate mean comparison test (Student's *t*-test for normally distributed data and Mann-Whitney U test otherwise).

We evaluated the effect of the intervention by comparing the data obtained from both groups. To do this, we first compared the characteristics of the intervention and control groups, the ERF of each group and the values of the anthropometric measurements (dependent variables) using the comparison tests appropriate for each case (t-student or Mann-Whitney U for continuous variables and the χ^2^ test for categorical variables). We also calculated Cohen's d index to measure the effect size in the comparison of the dependent variables.

To test the effect of the intervention at the global level, we performed a multiple linear regression analysis in which we took z-score BMI and body fat percentage at two years as dependent variables and ERF and the intervention group were incorporated as independent variables. We also analysed the accumulation of ERF in each intervention group, for which we performed a χ^2^ test on the resulting contingency table. We performed all calculations in the SPSS program, version 25.0.0 (IBM Corp, Armonk, NY, USA) and in R, version 4.2.0. (R Core Team, Vienna, Austria), taking α = 0.05 as the level of significance.

### Ethics

The trial presented little or no risk to participants and their offspring. Mothers gave written consent for the use of their data, with guaranteed rights, privacy, and integrity. We conducted the study following the Declaration of Helsinki to prevent and avoid risks to life and health. The regional Ethics Committee (Comité Coordinador de Ética de la Investigación Biomédica de Andalucía, CCEIBA) approved the study (Appendix I-II the **Online Supplementary Document**).

## RESULTS

We initially enrolled 135 mother-child pairs in the control group, with 109 remaining after loss to follow-up. The intervention group included a total of 145 cases, with 45 remaining after drop-out (**Figure 1**). There were no significant differences between either the maternal or neonatal variables between both groups (**Table 1**).

**Table 1:** Characteristics of the population studied

	Control (n = 109)	Intervention (n = 45)		
	**Mean (SD)**	**Mean (SD)**	**t**	***P*-value**
**Age in years**	32.37 (5.24)	32.93 (4.92)	-0.621	0.536
**Gestational age in weeks**	39 (1.33)	39.13 (1.83)	0.432	0.666
**Weight in kilograms**	3.31 (0.47)	3.22 (0.53)	1.079	0.282
**Length in centimeters**	49.81 (2.29)	49.51 (2.11)	0.748	0.455
	**n (%)**	**n (%)**	**χ^2^**	***P*-value**
**Parity**			0.381	0.944
1	46 (42.20)	20 (44.44)		
2	50 (45.87)	20 (44.44)		
>2	13 (11.93)	5 (11.12)		
**Sex**			0.238	0.625
W	58 (53.21)	22 (48.89)		
M	51 (46.79)	23 (51.11)		

SD – standard deviation

Concerning the comparative results of the ERF, the mean value of BMI at the beginning of gestation was similar between both groups, within the overweight range. We observed no significant differences in the percentage of pregnant women with GWG, the prevalence of DMG, the percentage of pregnant smokers, and the proportion cesarean delivery (**Table 2**).

**Table 2:** Prevalence of early risk factors comparison between groups and anthropometric characteristics of the offspring

	Control (n = 109)	Intervention (n = 45)		
**Prenatal**	**Mean (SD)**	**Mean (SD)**	***t***	***P*-value**
Pregestational BMI	26.52 (6.05)	26.47 (5.69)	0.052	0.958
	**n (%)**	**n (%)**	**χ^2^**	***P*-value**
GWG			0.251	0.616
*Adequate*	73 (66.97)	32 (71.11)		
*Excessive*	36 (33.03)	13 (28.89)		
Diabetes			1.385	0.239
*Yes*	8 (7.34%)	6 (13.33)		
*No*	101 (92.66)	39 (86.67)		
Smoking			0.007	0.935
*Yes*	14 (12.84)	6 (13.33)		
*No*	95 (87.16)	39 (86.67)		
Cesarean			1.627	0.202
*Yes*	27.52% (30)	8 (17.78)		
*No*	72.48% (79)	37 (82.22)		
**Postnatal**	**n (%)**	**n (%)**	**χ^2^**	***P*-value**
Initiation of breastfeeding	86 (78.97)	29 (64.44)	1.520	0.218
Exclusive breastfeeding at ≥4 mo*	49 (64.19)	19 (65.51)	0.016	0.898
Breastfeeding at ≥6 mo*	44 (44.95)	24 (51.11)	0.713	0.398
Early complementary feeding	1 (0.92)	0 (0.00)	0.416	0.519
Two-year sleep (10-12h)	97 (89.00)	41 (91.11)	0.154	0.695
RWG 0-4 mo	34 (31.19)	11 (24.44)	0.701	0.402
RWG 0-6 mo	38 (34.86)	11 (24.44)	1.593	0.207
RWG 0-12 mo	41 (37.61)	16 (35.55)	0.120	0.729
RWG 0-24 mo	49 (44.95)	22 (48.89)	0.089	0.765
	**Mean (SD)**	**Mean (SD)**	***t***	***P*-value**
Breastfeeding duration (months)*	7.56 (4.36)	9.34 (3.78)	-2.113	<0.05
Vitamin D (months)	5.56 (4.73)	9.24 (3.18)	-5.598	<0.001
z-score weight at birth	0.02 (1.02)	-0.20 (1.21)	1.12	0.26
z-score weight at four months	0.18 (1.01)	-0.19 (0.87)	2.11	<0.05
z-score weight at six months	0.38 (0.93)	0.19 (1.58)	0.91	0.36
z-score weight at one year	0.43 (0.95)	0.12 (0.96)	1.81	0.07
z-score weight at two years	0.50 (1.07)	0.48 (0.97)	0.11	0.91
Triceps fold	11.16 (2.05)	10.29 (1.86)	2.47	<0.001
Biceps fold	7.19 (1.95)	6.25 (1.37)	2.94	<0.001
Subscapular fold	6.85 (1.72)	6.41 (1.68)	1.47	0.14
Suprailiac fold	6.10 (1.66)	5.29 (1.19)	2.98	<0.001
z-score BMI at two years	0.19 (1.04)	0.08 (1.01)	0.60	0.55
Body fat in % at two years	19.33 (3.26)	17.63 (3.50)	2.87	<0.001

SD – standard deviation, BMI – body mass index, GWG – gestational weight gain, RWG – rapid infant weight gain

*Only cases that have started breastfeeding are included.

Regarding postnatal ERF, the duration of breastfeeding increased significantly after the intervention. Once breastfeeding exceeded the first month, we observed no significant differences in the percentage of exclusive breastfeeding ≥4 months or ≥6 months. Duration of vitamin D supplementation increased significantly in the intervention group. The percentage of children meeting the recommended hours of sleep elevated after the intervention, although not significantly. The percentage of children with RWG decreased at four, six, and 12 months and increased at 24 months, with non-significant differences.

**Figure 3:**
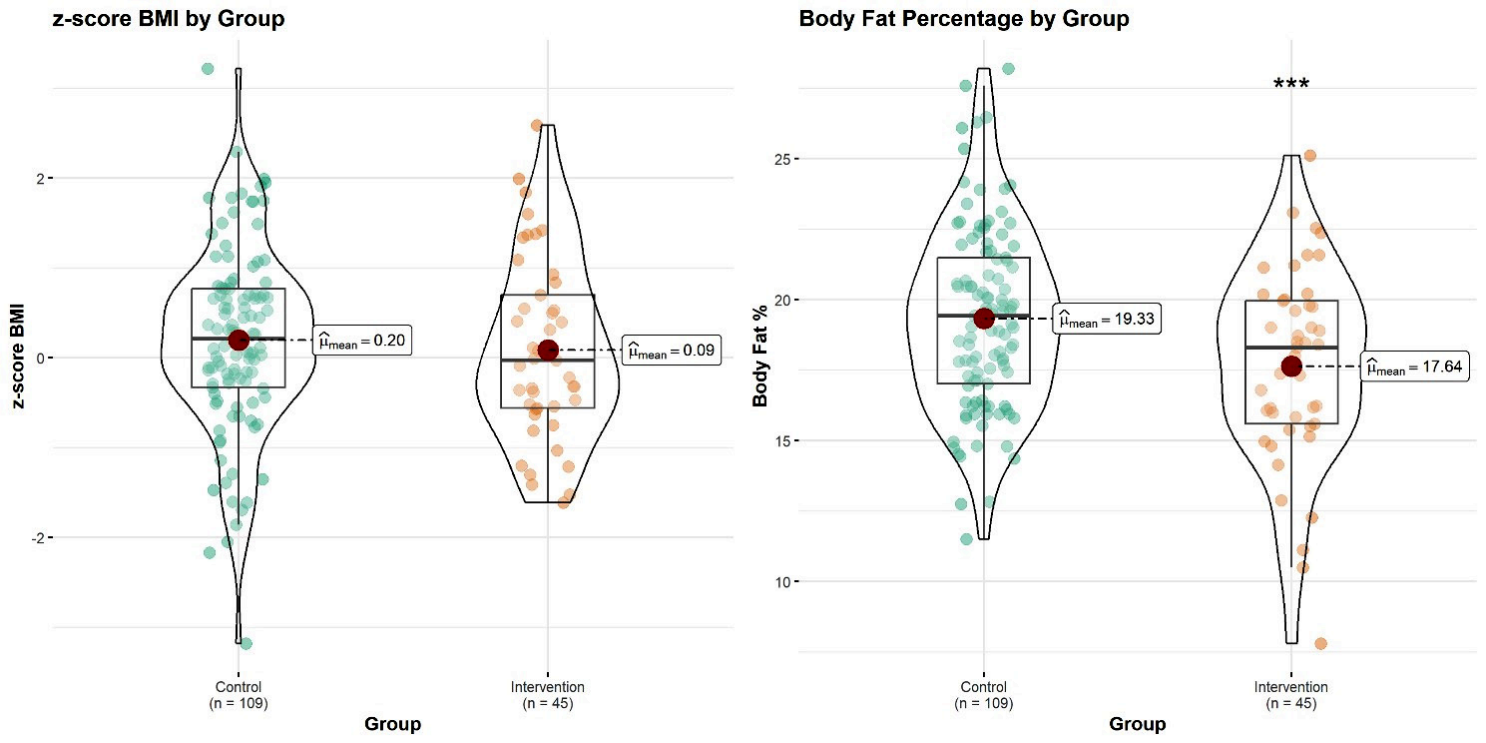
Violin plot of dependent variables.

Regarding anthropometric variables, the z-score weight was lower at birth and at four, six, and 12 months, but this difference disappeared at two years (**Table 2**). The skinfolds showed lower values in the intervention group, with triceps, biceps, and suprailiac ones being significant. The z-score BMI at two years was lower in the intervention group, although this difference was not statistically significant. However, the percentage of body fat at two years was significantly lower in the intervention group (**Figure 3**). Children in the intervention group had a 1.7% lower percentage of body fat compared to the control group.

The difference between the two groups concerning the variable body fat percentage at two years was significant, with a Cohen's d of 0.50, so the effect size is considered to be medium. The differences between the control group and the intervention group in terms of body fat percentage at two years are explained by the intervention by 24%.

To assess the overall intervention, we performed a multiple linear regression analysis with body fat percentage and z-score BMI as dependent variables (**Table 3**).

**Table 3:** Results of multiple linear regression analysis

	Body fat in %			z-BMI		
**Independent variable**	**β**	***t***	***P*-value**	**β**	***t***	***P*-value**
Pre-gestational BMI	0.154	1.703	0.091	0.097	1.103	0.273
GWG	0.191	2.107	<0.05	0.122	1.401	0.164
Diabetes	-0.050	-0.543	0.589	-0.146	-1.679	0.096
Smoking	0.275	3.041	<0.01	-0.021	-0.243	0.809
Cesarean	-0.013	-0.138	0.426	-0.018	-0.199	0.843
Breastfeeding duration	0.132	1.438	0.053	-0.025	-0.287	0.775
Vitamin D	0.184	1.897	0.426	-0.168	-1.931	0.056
Exclusive Breastfeeding ≥4 mo	0.036	0.399	0.053	0.000	-0.002	0.998
Breastfeeding ≥6 mo	0.075	0.799	0.426	-0.069	-0.783	0.435
RWG 0-24 mo	0.173	1.960	0.053	0.412	4.696	<0.001
group	-0.253	-2.815	<0.01	-0.169	-1.954	0.053

BMI – body mass index, GWG – gestational weight gain, RWG – rapid infant weight gain

The variable “group” (0 = control, 1 = intervention) was significantly associated with body fat percentage with β = -0.253, meaning that the children in the intervention group had significantly lower body fat percentage at two years. The relationship between groups and z-score BMI at two years was near to significance (*P* = 0.053).

Finally, we compared the accumulation of modifiable ERF (GWG, maternal smoking, breastfeeding <6 months, RWG 0-2 years, and inadequate sleep) (Appendix III in the **Online Supplementary Document**). The intervention group showed a higher percentage of cases accumulating no factors and a lower percentage accumulating 1 and 3 factors, although the difference was not significant.

## DISCUSSION

Our findings suggest that an intervention developed for preventing childhood obesity during the 1000-day period can modify body composition as early as two years of age. We developed our intervention on most of the known modifiable variables, through recommendations given during all health program visits and group workshops delivered throughout the intervention, focused on improvements in nutrition, physical exercise, habits, and lifestyles of the mother-child pair.

It is difficult to define which type of interventions are the most effective due to the variability between studies, both in the effect indicators, the duration and period of the intervention, the number of ERF, or the type of intervention. We can find the greatest effect in interventions focused on behavioral changes, ones aimed at diet and physical activity, ones including a greater number of ERF, and ones developed during the pre- and post-natal period [13].

Most of the interventions developed during this period evaluate their effect by BMI or z-score BMI at the end of the intervention, with some doing so several years later by directly determining overweight or obesity [33]. Comparison of the z-score BMI between the control and intervention groups at six to nine years would be a truer indicator of their effect on prevention. We evaluated the effect at the end of the intervention at two years of life, obtaining a decrease in z-score BMI in the intervention group, although the difference was not significant (0.19 (SD = 1.04) vs 0.08 (SD = 1.01); *P* = 0.55).

Few studies use adiposity indicators to assess the effect of interventions. The four skinfolds (biceps, triceps, suprailiac, and subscapular) showed lower values in the intervention group, with the first three differences being significant. A study developed birth and two years of age (discarding the gestation period) found no differences in skinfolds between intervention and control at two years [22]. In our study, body fat percentage was significantly lower in the intervention (19.33 (SD = 3.26) vs 17.63 (SD = 3.50); *P* < 0.001). To our knowledge, no studies used this indicator of an intervention’s effect during the whole 1000-day period.

Very few studies use both indicators (BMI and adiposity) to evaluate the effect of the intervention. It would be of interest to know which indicator is more sensitive for assessing the effect of interventions during the 1000-day period. Given that obesity is an increase in adiposity [34], it would seem more likely that a direct indicator of adiposity would be more sensitive than BMI, which has limitations in differentiating body fat from lean mass [35]. Moreover, several ERF have been shown to increase adiposity [36,37]. A study of body composition and breastfeeding in children under three years observed that BMI was not sensitive in detecting the effects identified by the triceps skinfold, which can be more sensitive to the differences that sometimes cannot be detected only by child-weight measurement [38]. In this sense, although our results show a decrease in both indicators, only the percentage of body fat was detected as significant.

The duration and period included in the intervention should be important for its effect. Most take place during gestation or after birth and a smaller number include both stages. Some studies cover gestation and some postnatal periods, up to six months [39] or one year [40]. Reducing the intervention period to within 1000 days means excluding some of the ERF associated with obesity, which appear from early gestation to the end of the second year. We found only one study that developed the intervention during a period similar to ours, from before week 15 to the end of the second year of life [41], which found no significant differences in BMI at two years, despite a significant improvement in GWG during gestation.

Regarding the number of ERF, most studies did not find an effect on weight or body composition when they considered a single factor such as sleep [42], duration of breastfeeding [43], physical activity of the children [44], or GWG [41], although they improved the results of the intervening variable. Our intervention addressed most potentially modifiable ERF associated with childhood obesity in the literature [7], as we considered doing so vital. In the previous analysis of our control group, we found that the accumulation of factors in the same individual increased the z-score BMI and body fat percentage at two years [14], similar to other studies with the relative risk of obesity at six years [45].

Some studies assessed the effect of interventions indirectly by evaluating the decrease in the prevalence of ERF. We only found significant improvements in breastfeeding duration (7.56 (SD = 4.36) vs 9.34 (SD = 3.78), *t* = -2.11; *P* = 0.039), and in months of vitamin D supplementation during the first year (5.57 (SD = 4.73) vs 9.24 ± 3.18), *t* = -5.60; *P* < 0.001). Full breastfeeding at >6 months was found to be associated with lower childhood (5-6 years) fat mass [46]. Another study showed an inverse association between breastfeeding duration and skinfold thickness in children younger than three years [38]. This association may be because higher protein intake in formula-fed infants contributes to higher plasma insulin that stimulates greater fat deposition [47]. Moreover, children who had higher breast milk intake in early life had more favorable leptin concentrations [48]. A recent study [49] has shown that the duration of breastfeeding ≥6 months only has an indirect effect on the risk of overweight or obesity in childhood, through the reduction of the risk of rapid weight gain. Several studies have demonstrated the relationship of vitamin D with body composition in childhood. Lower Vitamin D level in the third trimester of pregnancy was associated with higher fat mass at four to six years of age [50]. Another study found an association between higher serum vitamin D levels at three years and lower fat mass composition after supplementation during the first year [51]. Although this relationship is not yet clear, this association between deficient serum vitamin D and higher fat mass should be an argument to reinforce the recommendation of supplementation during the first year.

Meanwhile, the intervention failed to improve the results of a greater number of modifiable ERF of obesity: GWG, RWG from birth to two years, smoking, exclusive breastfeeding ≥4 months or increased hours of sleep at two years. However, adiposity indicators at the end of the intervention have significantly improved. Furthermore, when analysing the differences in the total sample jointly through regression, including all the variables to see the influence of each one, we determined that the group, taken as one more variable, has a significant effect on the percentage of body fat at two years. This confirms that belonging to the intervention group has a positive effect on fat percentage, suggesting that the intervention was effective.

It may be surprising that, without significant improvements after intervention in several ERF, we obtained a decrease in fat percentage. This could be explained by two factors. First, the intervention group presented a higher percentage of cases that did not accumulate any factor and a lower percentage of accumulation of one and three factors, with the accumulation of ERF related to higher values of fat mass [19]. Second, the intervention additionally provided recommendations related to habits and lifestyles that begin after birth (breastfeeding, taste preferences, satiety regulation, etc.). The effect of this type of multifactorial intervention may not be reflected in the differences in each of the modifiable variables, but in the z-score BMI and body fat percentage at two years, proposed as indicators.

These findings confirm that childhood obesity is multifactorial and that is necessary interventions during gestation and the following two years. Our intervention is limited in time to the first 1000 days, and other factors may have influence after two years of age. However, adequate metabolic programming at the end of this period and initiating the acquisition of healthy habits at early ages seem to have a protective effect against obesogenic factors at later ages.

Regarding the strengths of our study, it is, to the best of our knowledge, one of the first to analyse the effect of a multifactorial intervention that encompasses the entire 1000-day period, relating this intervention to anthropometric measurements. It is also one of the few studies to analyse the percentage of body fat as an effect indicator.

However, it also has two main limitations: non-randomisation and the sample size of the intervention group. Randomisation has not been possible because parents have the freedom and the right to choose the pediatrician. However, we had a homogeneous population, which reduced the risk of bias.

Moreover, despite the COVID-19 pandemic leading to significant losses in the intervention group sample, this has not limited our statistical power. Moreover, we know the influence that the COVID-19 pandemic and confinement have in the life habits of the child and adolescent population, which could have possibly biased our findings. Specifically, in children under two years of age, there is a change in lifestyle habits, with an increase in screen time, consumption of unhealthy snacks and sedentary habits, although some habits improved, such as more frequent family meals and more hours of sleep [52].

## CONCLUSIONS

Body fat percentage showed a significant decrease in the intervention group at the end of the intervention, at the age of two years. Follow-up of both groups until the age of six to eight years will be able to measure, using z-score BMI, the impact of the intervention on the ultimate goal of reducing the prevalence of childhood obesity, and determine whether either or both of the proposed indicators (body fat percentage and z-score BMI) are valid early indicators of risk for childhood obesity. However, for assessing the effect of interventions during the 1000-day period, body fat percentage was shown to be a better indicator than z-score BMI.

However, the significant effect of the intervention observed on the adiposity indicator at two years did not correlate with the low impact observed on most of the ERF assessed. We consider that the intervention developed a global effect as it included a high number of ERF and took place over the entire 1000-day period, offering a set of recommendations on lifestyle, sleep, diet and physical activity, throughout the period, in all visits and workshops where families were contacted. This overall effect also reduced the number of ERF accumulated in the same individual, which would contribute to reducing the risk of obesity in later life. Therefore, we believe that the effectiveness of an obesity prevention intervention should not be assessed based on the effect on individual risk factors, but on final outcomes.

In the coming years, the most effective primary prevention interventions for childhood obesity should be initiated at the earliest stages of development, include as many ERF as possible, and cover a long period of time – if possible the entire 1000-day period.

## Additional material


Online Supplementary Document
