# Supplementary material for: Impact of an early 1000-day intervention for obesity prevention on adiposity and BMI at two years of age: A quasi-experimental study
Source: J Glob Health. 2023 Dec 13;13:04145. doi: 10.7189/jogh.13.04145 (PMC10715455; doi:10.7189/jogh.13.04145)
Supplement: Online Supplementary Document [file jogh-13-04145-s001.pdf]

CONSENTIMIENTO INFORMADO POR ESCRITO

Título del estudio: Evaluación de la efectividad de una intervención para la prevención de la obesidad infantil, desarrollada en atención primaria durante el embarazo y dos primeros años de vida, sobre la composición corporal a la edad de dos años.
Promotor: Universidad de Cádiz

Yo (nombre y apellidos) ______________________________________

He leído y comprendido la hoja de información que se me ha entregado.

He podido hacer preguntas sobre el estudio.

He recibido suficiente información sobre el estudio.

He hablado con:

____

(nombre del investigador)

Comprendo que mi participación es voluntaria.

Comprendo que puedo retirarme del estudio:

1º Cuando quiera

2º Sin tener que dar explicaciones.

3º Sin que esto repercuta en mis cuidados médicos.

Presto libremente mi conformidad para participar en el estudio dentro del grupo de intervención.

FECHA: FIRMA DEL PARTICIPANTE

CONSENTIMIENTO DEL REPRESENTANTE

Título del estudio: Evaluación de la efectividad de una intervención para la prevención de la obesidad infantil, desarrollada en atención primaria durante el embarazo y dos primeros años de vida, sobre la composición corporal a la edad de dos años.
Promotor: Universidad de Cádiz.

Yo (nombre y apellidos) ____ en calidad de _________________ (relación con el participante) de ____ (nombre del participante en el estudio)

He leído y comprendido la hoja de información sobre el estudio.

He podido hacer preguntas sobre el estudio.

He recibido respuestas satisfactorias a mis preguntas

He recibido suficiente información sobre el estudio.

He hablado con:

. _____________________________________

(nombre del investigador)

Comprendo que la participación es voluntaria.

Comprende que puede retirarse del estudio:

1º Cuando quiera

2º Sin tener que dar explicaciones.

3º Sin que esto repercuta en sus cuidados médicos.

Y presto mi conformidad con que ____ (nombre del participante en el estudio) participe en este estudio dentro del grupo de intervención.

FECHA: FIRMA DEL REPRESENTANTE

DICTAMEN ÉTICO DEL COMITÉ COORDINADOR DE ÉTICA DE LA INVESTIGACIÓN BIOMÉDICA DE ANDALUCÍA

D. Joaquín Alanís López, Secretario del
Comité Coordinador de Ética de la Investigación Biomédica de Andalucía

CERTIFICA

Que este Comité, ha evaluado en los aspectos éticos, el estudio de investigación perteneciente a la convocatoria de subvenciones para la financiación de la I+D+i biomédica y en ciencias de la salud en la provincia de Cádiz, con titulo: **"Evaluación de la efectividad de una intervención para la prevención de la obesidad infantil, desarrollada en atención primaria durante el embarazo y dos primeros años de vida, sobre la composición corporal a la edad de dos años."** y cuya investigadora principal es DOÑA MERCEDES DÍAZ RODRÍGUEZ.

Y considera que:

Se cumplen los requisitos necesarios de idoneidad del protocolo con relación a los objetivos y se ajusta a los principios éticos aplicables a este tipo de estudios y recogidos en la Declaración de Helsinki de 1964, de la Asociación Médica Mundial, y enmiendas posteriores, y en el Convenio del Consejo de Europa de 1996, relativo a los Derechos Humanos y a la Biomedicina,.

Según la información aportada en el manuscrito el tratamiento de los datos de carácter personal de los participantes se ajusta a lo dispuesto en la Ley Orgánica 15/1999 de 13 de diciembre de protección de datos de carácter personal.

Están justificados los riesgos y molestias previsibles para los participantes. Es adecuado el procedimiento para obtener el consentimiento informado.

Por todo lo anterior:

El *Comité Coordinador de Ética de la Investigación Biomédica de Andalucía,* en su reunión del día 26 de abril de 2018 (Acta 04/2018) tras la evaluación ética del citado estudio acuerda emitir un **DICTAMEN FAVORABLE**

Lo que firmo en Sevilla, a 2 de julio de 2018

Joaquin Alanis López

Avda. de la Innovación, s/n. Edificio Arena 1. Apdo. Correos 17.111. 41080 Sevilla
Teléf. 95 500 63 00. Fax 95 540 72 02

Código Seguro De Verificación:	z8Xq22BdFreCboKMQhFqVQ==	Fecha	02/07/2018
Normativa	Este documento incorpora firma electrónica reconocida de acuerdo a la Ley 59/2003, de 19 de diciembre, de firma electrónica.		
Firmado Por	Joaquin Alanis Lopez		
Url De Verificación	https://ws069.juntadeandalucia.es/verifirma/code/z8Xq22BdFreCboKMQhFqVQ==	Página	1/2

Anexo I

COMPOSICION DEL CEI

Presidente	Demetrio Mariano Aguayo Canela
Vicepresidente	Fernando Antúnez Estévez
Secretaria técnica	Joaquin Alanís López
Vocales	María Teresa Aldabó Pallás
	Macarena Anchóriz Esquitino
	Miguel Angel Arrabal Polo
	Reyes Bernabé Caro
	Encarnación Blanco Reina
	Francisco Bombillar Saénz
	Juan María Casado Salinas
	Celia Castillo Valverde
	Juan Jesús Cobacho de Alba
	Cristina Lucia Dávila Fajardo
	Alberto Delgado García
	Miguel Delgado Rodríguez
	Sandra Flores Moreno
	Isabel García Ríos
	Jesús Gómez Mateo
	Daniel Palma Morgado
	Enrique Raya Álvarez
	Mónica Saldaña Valderas
	Mercedes Sánchez Lanuza Rodríguez
	Jaime Torelló Iserte
	Matilde Vera Rodríguez
	José Mª Villagrán Moreno

Avda. de la Innovación, s/n. Edificio Arena 1. Apdo. Correos 17.111. 41080 Sevilla
Teléf. 95 500 63 00. Fax 95 540 72 02

Código Seguro De Verificación:	z8Xq22BdFreCboKMQhFqVQ==	Fecha	02/07/2018
Normativa	Este documento incorpora firma electrónica reconocida de acuerdo a la Ley 59/2003, de 19 de diciembre, de firma electrónica.		
Firmado Por	Joaquin Alanis Lopez		
Url De Verificación	https://ws069.juntadeandalucia.es/verifirma/code/z8Xq22BdFreCboKMQhFqVQ==	Página	2/2

Appendix III. ERF accumulation in both groups.

Factors	Control (n=109)	Intervention (n=45)
**0**	15 (13.76%)	10 (22.22%)
**1**	39 (35.78%)	13 (28.89%)
**2**	38 (34.86%)	16 (35.55%)
**3 or more**	17 (15.60%)	6 (13.33%)

$chi^2 = 7.752$, p-value = 0.101
